# Supplementary material for: Learning curve and surgical outcome of robotic assisted colorectal surgery with ERAS program
Source: Sci Rep. 2022 Nov 29;12:20566. doi: 10.1038/s41598-022-24665-w (PMC9709162; doi:10.1038/s41598-022-24665-w)
Supplement: Supplementary file 2 — Supplementary Information 2. [file 41598_2022_24665_MOESM2_ESM.zip › RAL Raw data-Ñ[▒K/067/Patho_ñw╝╨░O▒Kñσ.pdf]

臺中榮民總醫院 臺中市臺灣大道四段1650號 病理部活體組織切片 臨床醫學實驗及病理診斷中心 檢驗(查)報告

[redacted]

Pathologic diagnosis:
1. Sigmoid colon, robotic anterior resection --- a. Status post polypectomy at other hospital with foreign-body type giant cell reaction and chronic inflammation. No residual tumor found. The bilateral cut ends and mesocolic lymph nodes (0/12) are unremarkable.
b. Hyperplastic polyps.
2. IMA root, dissection --- Unremarkable fibroadipose tissue.
3. Colon, cut end, distal, resection --- Chronic inflammation.

Gross description:
The specimen consists of 1) a segment of colon, 10.5 cm in length and 3 cm in proximal cut end circumference, 3.7 cm in distal circumference, and mesocolic soft tissue. There is area of erythromatous change, labeled with clip, noted over the mucosa, with the distance of 2.8 cm to the nearest cut end. There are several smll polpyoid lesions, up to 0.2x0.2 cm, over the mucosa. On cross section, no invasive mass lesion is identified grossly. The both cut ends are free. 2) IMA root, a piece of tan yellow soft tissue, 2.5x1.8x0.7 cm. 3) distal cut end, a piece of tan mucosa-covered soft tissue, 2.4x2x1.3 cm. Representative part for section: A) proximal cut end B) distal cut end C-E) colon tissue around the clip (inked with green dye) F-G) colon polyps H-J) specimen 2-3 K-U) lymph nodes.
#T-59300_2 #M-09413_2 0 1105 SP0000
#T-59300_2 #M-72042_2 0 1105 000000
#T-C4560_2 #M-09183_2 0 1105 000000

已電子簽章

警告：本單僅供醫師參考使用，不具任何申請效力！！
請病患妥為保管，若不慎遺失務自負個人資料外洩責任！
